# On Compton scattering as a source of background in coherent diffraction imaging experiments

**DOI:** 10.1107/S1600577521000722

**Published:** 2021-02-18

**Authors:** Oier Bikondoa, Dina Carbone

**Affiliations:** a Department of Physics, University of Warwick, Gibbet Hill Road, Coventry CV4 7AL, United Kingdom; b XMaS – The UK Materials Science Facility, ESRF – The European Synchrotron, CS40220, F-38043 Grenoble Cedex 09, France; c MAX IV Laboratory, Fotongatan 2, 225 94 Lund, Sweden

**Keywords:** coherence, imaging, scattering, X-rays

## Abstract

Compton scattering is generally neglected in diffraction experiments because the incoherent radiation it generates does not give rise to interference effects and therefore is negligible at Bragg peaks. However, as the scattering volume is reduced, the difference between the Rayleigh (coherent) and Compton (incoherent) contributions at Bragg peaks diminishes and the incoherent part may become substantial. The consequences can be significant for coherent diffraction imaging at high scattering angles: the incoherent radiation produces background that smears out the secondary interference fringes, affecting thus the achievable resolution of the technique. Here, a criterion that relates the object shape and the resolution is introduced. The Compton contribution for several object shapes is quantified, and it is shown that the maximum achievable resolution along different directions has a strong dependence on the crystal shape and size.

## Abstract 2

How Compton scattering can reduce the real space resolution achievable with the Bragg coherent diffraction imaging technique is analysed.

## 1. Introduction   

In the last decade, the use of the coherence properties of X-rays produced at third-generation synchrotron radiation sources has markedly increased. Techniques exploiting coherence have been developed and employed in different ways to study the morphology and strain of materials using inverse microscopy approaches, or the dynamics, using photon correlation spectroscopy (Nugent, 2010 ▸). For these techniques, markedly for inverse microscopy, coherence propagation and preservation are very important issues, as they can affect in a strong way the inversion’s outcome. Partial coherence, as widely discussed in the literature (Sinha *et al.*, 1998 ▸; Whitehead *et al.*, 2009 ▸; Nugent, 2010 ▸), washes out interference effects and thus compromises the inversion process or the achievable spatial resolution. For this reason, the use of coherence preserving optics is especially important (Paganin, 2006 ▸). The propagation of the X-ray beam through optical components of hard X-ray beamlines and the apparent loss of coherence sometimes observed has also been discussed (Vartanyants & Robinson, 2003 ▸; Nugent *et al.*, 2003 ▸). It has been argued that, in the absence of vibrations or instabilities, the coherence properties of the radiation cannot be degraded during the propagation through optical components (Nugent *et al.*, 2003 ▸). This is justified in terms of conservation of the phase space and considers that the experimental X-ray system is closed, without external influences (Nugent, 2010 ▸). Finally, special attention has also been given to the characterization of the incoming wavefront to disentangle the contributions of the incoming wavefield and the sample scattering function (Kewish *et al.*, 2010 ▸; Schropp *et al.*, 2010 ▸; Mastropietro *et al.*, 2011 ▸; Björling *et al.*, 2020 ▸). However, even in the absence of external influences or vibrations, the coherence may be degraded via the interaction with the sample through quantum effects such as Compton scattering, in which the incoming photon transfers part of the momentum and energy to electrons. The Compton scattered photons lose their phase relationship with the incoming photons.

Notwithstanding, Compton scattering can often be ignored. In conventional X-ray crystallography, the angles and intensities of the beams diffracted by a crystal are measured. Due to the periodic arrangement of the atoms or molecules within the crystal into a space lattice, diffraction only occurs at very specific directions, for which the spherical wavelets scattered by each lattice point interfere constructively. These directions are determined by Bragg’s law. For a crystal with *N* lattice points illuminated with a monochromatic X-ray beam, at those points satisfying Bragg’s law, in the kinematical approximation the maximum intensity is proportional to the square of the total number of lattice points, *i.e.* *I*_coh_ ∝ *N* ^2^ (James, 1965 ▸). We include the subscript ‘coh’ to stress that interference effects can only arise from the coherently scattered photons (Rayleigh scattering). The contribution due to Compton scattering, being incoherent, only contributes as *I*_inc_ ∝ *N* and the ratio of the incoherent/coherent contribution scales as 1/*N*. Already for small crystals with few thousands lattice points, the incoherent contribution is insignificant with respect to the coherent part. This is why Compton scattering is usually neglected in X-ray diffraction experiments on single crystals, even if the total scattering cross section of Compton scattering can already be of the same order of magnitude as that of Rayleigh (*i.e.* elastic) scattering for low-*Z* elements in the 10–20 keV range.

In Bragg coherent diffraction imaging (BCDI) and Bragg ptychography the intensity distributions at, and around, Bragg peaks, that is, the intensity at the Bragg positions as well as the secondary (or subsidiary) interference fringes, are employed to obtain a high-spatial-resolution image of the diffracting crystal with high strain sensitivity (Stagl *et al.*, 2014 ▸). These subsidiary maxima are much weaker than the principal – Bragg – maximum and their extent (*i.e.* number of visible or measurable fringes) limits the achievable spatial resolution. Compton scattering, as a process that increases the background and reduces the fringe visibility, may lessen the maximum resolution that can be obtained in the reconstructions because it affects the signal-to-noise ratio, as we shall discuss below. This is particularly important when the signal is weak, which is generally the case of BCDI experiments. With the increased coherent flux provided by modern sources, BCDI has great potential for the study of smaller particles. In the case of catalytic or electrochemical reactions (Richard *et al.*, 2017 ▸; Rochet *et al.*, 2019 ▸; Björling *et al.*, 2019 ▸), particle sizes in the 1–10 nm range useful for industrially relevant reactions (Campbell *et al.*, 2016 ▸) can be studied. The relative intensity between the Bragg peaks and the secondary maxima depends on the particle size; thus, the relative effect of Compton scattering will be more important for small particles. Furthermore, in electrochemistry experiments, high energies can be beneficial to penetrate through the electrolyte. The increased brilliance of the fourth-generation synchrotron radiation sources, *i.e.* the diffraction-limited storage rings (DLSRs), may generate a 100-fold increment in coherent flux and consequently facilitate experiments that require both high energies and large coherent flux. However, increasing the energy has the side effect of enhancing the Compton scattering, which scales with the energy (see Section 2.1). It is therefore of interest to evaluate the Compton scattering component and estimate its effect on a coherent diffraction experiment, especially on the signal-to-noise ratio.

In this article, we discuss the relevance of Compton scattering in BCDI experiments and quantify it. We start from the fundamentals (Section 2): we first review the coherent and incoherent scattering by an electron (Section 2.1) and an atom (Section 2.2) and generalize it for a small crystal (Section 2.3). In Section 3 we analyse how the presence of a background affects the interference fringes and we obtain a criterion that relates the shape function of the scattering object and the differential cross sections of coherent and incoherent scattering. Some practical examples are examined in Section 4. We discuss in Section 5 some of the implications of our results and we end with the conclusions (Section 6).

## 2. Coherent and incoherent scattering   

We first review the basic formulas of the X-ray scattering theory for an electron and an atom and use them to assess the incoherent and coherent contributions scattered by a small crystal.

### 2.1. Scattering by an electron   

In 1923, Compton interpreted the shift in wavelength that had been observed when X-rays are scattered by atoms in terms of particle-like collisions between an incoming photon and the atom’s electrons (Compton, 1923 ▸). A few years later, Klein and Nishina developed a quantum-mechanical model and obtained the cross section of the Compton scattering process for a free electron (Klein & Nishina, 1929 ▸). For linearly polarized incoming radiation – which is the most relevant case for synchrotron-based experiments, as synchrotron radiation is mostly polarized in the plane of motion of the electrons in the storage ring – the Klein–Nishina (KN) formula reads (Klein & Nishina, 1929 ▸; Evans, 1958 ▸)
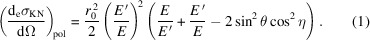
Here, 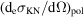 is the differential KN cross section of an incoming linearly polarized photon scattered per electron into the differential solid angle dΩ in the direction defined by the angles θ and η, and summed over all possible polarizations of the scattered photon. θ is the scattering angle, *i.e.* the angle between the wavevectors of the incident and scattered radiation, which defines the scattering plane (note that here we follow the convention used in the scattering community rather than the one used in diffraction, where the scattering angle is θ = 2Θ). η is the angle between the electric field vector of the incoming radiation and the scattering plane. *r*_0_ and ɛ = *E*/*m*_e_ *c* ^2^ are the classical electron radius and the photon energy in units of the electron rest-mass (*m*_e_) energy, respectively. The KN cross section describes the Compton interaction, which is inelastic: part of the incoming photon energy (*E*) is transferred to the electron and the Compton scattered photons have lower energy (*E*′). The Compton equation relates *E* and *E*′,
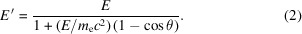
Using equation (2), the KN differential cross section can be expressed as a function of the incoming photon energy (*E*) only,
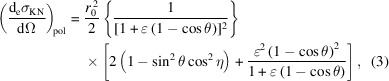
where 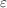 = 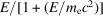. For X-ray energies *E* ≃ 10–100 keV the energy of the incoming photons cannot be neglected. However, as 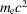 = 0.511 MeV, the approximation 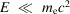 can be used to expand equation (3) into a Maclaurin series of ɛ. Retaining only the first-order term in ɛ,
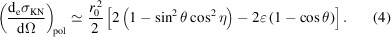
The term proportional to ɛ in the square brackets of equation (4) embodies the inelastic scattering contribution to the KN scattering cross section and is proportional to the energy of the incoming photons. The left term, independent of ɛ, accounts for the elastic scattering. Equation (4) distinctly shows that the differential scattering cross section is smaller than what would be expected from purely elastic scattering and that for higher energies the total cross section diminishes while the inelastic contribution increases with respect to the elastic part.

The KN differential cross section for unpolarized incoming radiation is obtained from equation (3) considering an incoming beam with two linearly polarized components, each one carrying half of the total intensity,
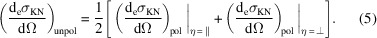
This yields
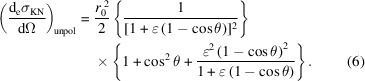
The same result is obtained from equation (3) averaging over all possible η directions: 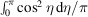 = 1/2.

The Thomson scattering cross section is the low-energy limit of the KN cross section of a free electron and it is obtained when the energy of the incoming photons *E* is much smaller than the electron rest energy 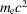 and can be ignored,
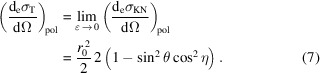
In particular, for unpolarized incoming radiation, 
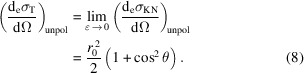
In the following sections, we drop the pol/unpol subscript from the KN and Thomson differential cross sections, with the implicit assumption that the adequate formulas are used for each case.

### 2.2. Scattering by an atom   

**Figure 1:**
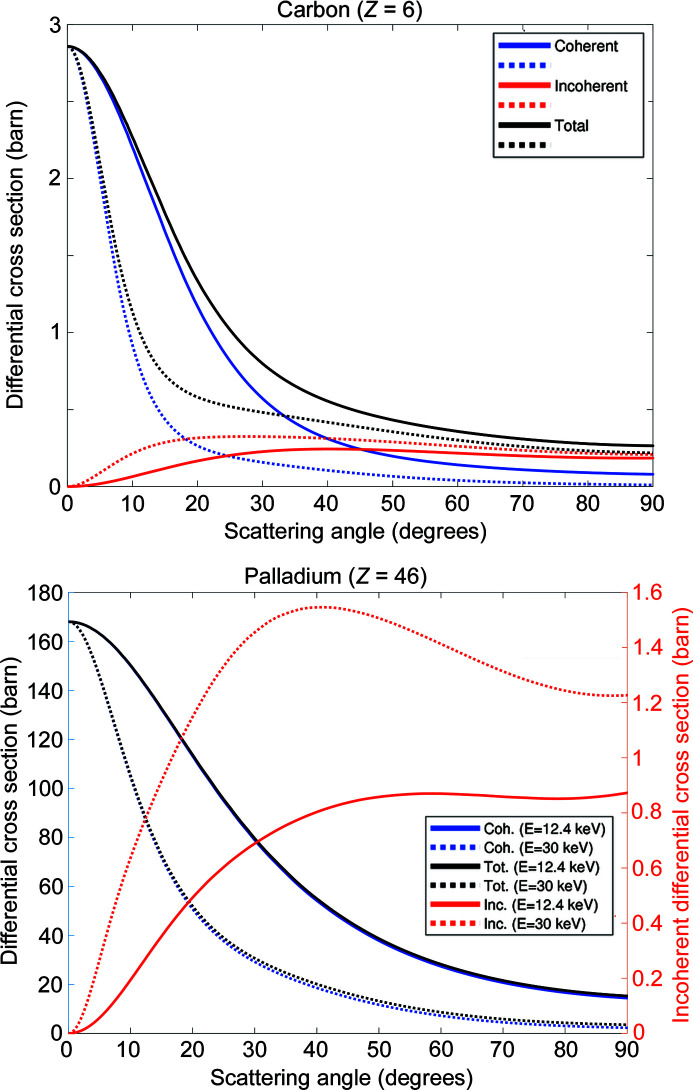
Differential (with respect to solid angle) cross sections at energies *E* = 12.4 keV (solid lines) and *E* = 30 keV (dotted lines): coherent (blue), incoherent (red), total (black) scattering. Carbon (top), palladium (bottom). For clarity, the palladium differential cross sections are plotted using two *Y*-axes, as the difference between the coherent and incoherent cross sections is large.

**Figure 2:**
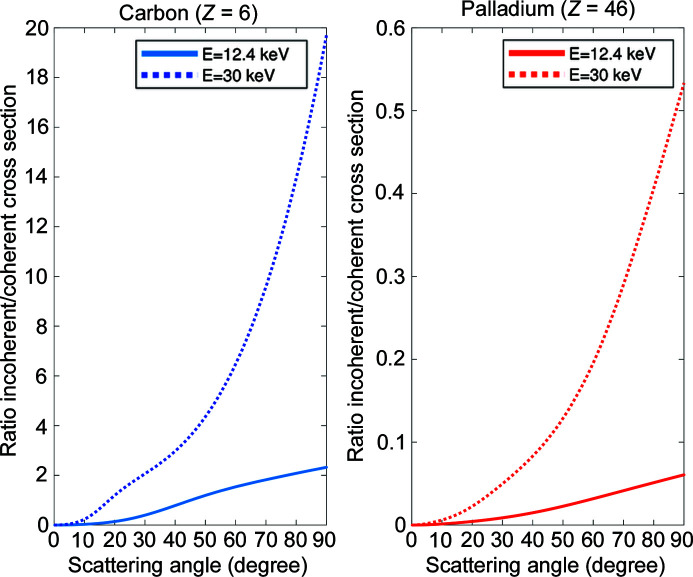
Ratio between the differential incoherent and coherent scattering cross sections for carbon (left) and palladium (right) at energies *E* = 12.4 keV (solid) and *E* = 30 keV (dotted).

In the case of atoms, the momentum and binding energies of the electrons have to be taken into account to calculate the scattering cross section. The atomic cross section can be written as a sum of two terms (Veigele *et al.*, 1966 ▸): (1) a term accounting for coherent scattering (Rayleigh scattering) in which after the scattering process the atom is left in its initial state and no energy is absorbed; (2) a term accounting for incoherent scattering (Compton scattering), in which energy as well as momentum is transferred and the phase relationship between the incoming and scattered radiation is also lost,
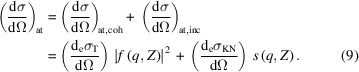
The coherent term is proportional to the differential Thomson scattering cross section for a free electron, 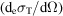 [equation (7)] – which is independent of the energy – and the square of the atomic form factor *f*(*q*, *Z*). The incoherent term is proportional to the differential KN scattering cross section, 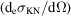 [equation (3)] and the incoherent scattering function *s*(*q*, *Z*). The limits of these functions for small and large *q* momentum transfer values are: *f*(0, *Z*) = *Z*, *f*(∞, *Z*) = 0, *s*(0, *Z*) = 0 and *s*(∞, *Z*) = *Z* (Veigele *et al.*, 1966 ▸; Hubbell *et al.*, 1975 ▸). Here we use lower-case letters *f* and *s* for the coherent and incoherent atomic form factors, respectively, to avoid confusion with the crystal structure factor introduced in Section 2.3. The most usual convention in the literature discussing the atomic form factors is to use upper-case letters *F* and *S* (Veigele *et al.*, 1966 ▸). Tabulated values of *f*(*q*, *Z*), *s*(*q*, *Z*) and 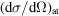 calculated using different models can be found in the literature (Hubbell *et al.*, 1975 ▸). Programs to calculate the cross sections are also available (Fernandez *et al.*, 2011 ▸). We have used the *SAP* program (Fernandez *et al.*, 2011 ▸) to calculate the atomic form factor and the incoherent scattering functions of carbon and palladium at 12.4 and 30 keV and obtain the Rayleigh and Compton scattering cross sections. The differential cross sections are shown in Fig. 1 ▸ and the ratios of Compton to Rayleigh scattering in Fig. 2 ▸. In the case of carbon, for scattering angles smaller than 45°, the Rayleigh differential cross section dominates. However, above ∼45° the inelastic scattering becomes more important than the elastic.

### 2.3. Scattering by a small crystal   

The total intensity scattered by a crystal will have contributions due to incoherent and coherent scattering, *I*_tot_ = *I*_inc_ + *I*_coh_. In the same way as done for the scattering by an atom, the total scattered intensity by the crystal can be divided into a coherent and an incoherent part,
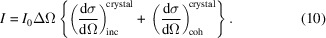
The coherent part will be proportional to the Thomson scattering cross section and will depend on the relative positions of the scatterers and their number. The incoherent part will be proportional to the KN cross section and will only depend on the total number and type of scatterers (*i.e.* not on their position). These coherent and incoherent terms are derived in Sections 2.3.1 and 2.3.2, respectively.

#### 2.3.1. Coherent scattering   

The (coherent) scattering by a small crystal was first treated by von Laue (1936 ▸) and shortly after by Patterson (1939 ▸) and Ewald (1940 ▸). The electronic density of an infinite crystal can be expressed as the convolution of the electronic density of the unit cell, ρ_0_(**r**), and an infinite three-dimensional lattice, 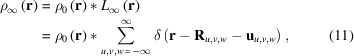
with **R**_*u*,*v*,*w*_ = *u* **a** + *v* **b** + *w* **c**, where **a**, **b**, **c** are the vectors defining the unit lattice, 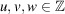 are integer numbers and the vectors 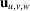 account for small distortions of the lattice from their ideal positions. In equation (11), it has been assumed that the lattice distortions do not affect the unit cell; that is, the effect of the distortions is to displace the unit cells as a rigid body, which is the so-called Takagi approximation (Takagi, 1969 ▸; Pietsch *et al.*, 2004 ▸). This assumption does not change the main conclusions of our study and simplifies the analysis. The more general case of small distorted crystals with several atoms in the unit cell is treated by Krivoglaz (1996 ▸) and is also discussed by Vartanyants & Robinson (2001 ▸).

For an infinite crystal illuminated with a plane monochromatic wave, the wavefield or complex amplitude scattered in the far field, and considering that the Born first approximation is valid, is proportional to the Fourier transform (FT; 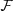) of its electronic density,
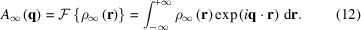
In this study, we can adopt the first Born approximation because we shall consider small crystallites for which multiple scattering is negligible. The finite size of a crystal with shape Γ is considered by introducing a shape function ϕ_Γ_(**r**) that fulfils
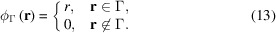
That is, ϕ_Γ_(**r**) equals 1 if the position vector is inside the crystal and 0 if it lies outside it. The electronic density of the finite crystal is expressed as 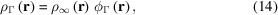
and, using the convolution theorem, its Fourier transform is 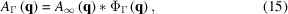
where * denotes the convolution operation and 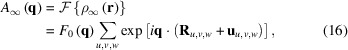
in which *F*_0_(**q**) is the scattering amplitude of the unit cell or structure factor,
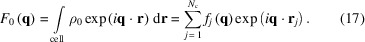
The integration extends only to the unit cell, and in the case of a unit cell composed of *N*_c_ individual atoms it can be replaced by a summation, *f*_*j*_(**q**) being the coherent atomic scattering factor of the atom at position *r*_*j*_ in the unit cell and with the summation index *j* extending over all the atoms in the unit cell. The summation term in equation (16) is over the lattice points.

In equation (15), 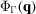 = 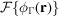 is the FT of the shape function,
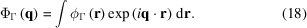
In the absence of lattice distortions, all 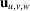 = 0 and equation (15) can be written in terms of the Dirac delta function (Giacovazzo *et al.*, 1992 ▸),
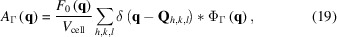
with 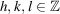 and **Q**_*h*,*k*,*l*_ = *h* **a** ^*^ + *k* **b** ^*^ + *l* **c** ^*^ being the generic vector of the reciprocal space and 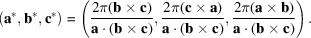

*V*_cell_ = **a**·(**b** × **c**) is the volume of the lattice unit cell.

Thus, the scattering amplitude *A*_Γ_(**q**) is a function which consists of the repetition of the FT of the shape function at every reciprocal lattice node, weighed by the structure factor *F*_0_(**q**). As the shape function ϕ_Γ_ is real-valued, its FT, Φ_Γ_, is centro-symmetrical about each reciprocal lattice point (Ewald, 1940 ▸).

Finally, the diffracted intensity will be proportional to the square of the scattered amplitude *A*_Γ_(**q**), and, neglecting small cross terms, it can be written approximately as
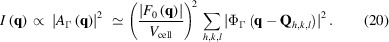
Close to a particular reciprocal space point 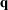 = 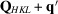, with 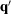 small,
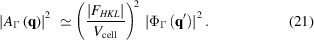

*F*_*HKL*_ is the structure factor for the reflection (*H*, *K*, *L*). The coherent differential cross section is thus 
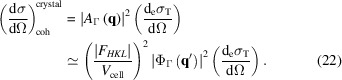
The (coherent) atomic form factor in equation (9) enters into equation (22) through the structure amplitude of the unit cell, |*F*_*HKL*_| in equation (21). The maximum of 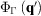 occurs when 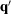 = 0 and is equal to the volume of the whole crystal: 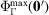 = 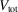. The total volume is the integration of the shape function 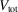 = 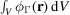. Equation (22) can be written in terms of a normalized FT shape function, 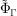 = 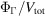,
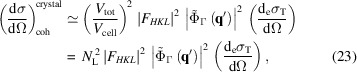
where 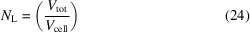
is the total number of unit cells or lattice points in the crystal.

#### 2.3.2. Incoherently scattered intensity   

The intensity scattered incoherently by a crystal will only depend on the number of scatterers and their incoherent scattering function *S*_*j*_(**q**, *Z*). If the unit cell contains *N*_c_ atoms, the crystal incoherent scattering function is 
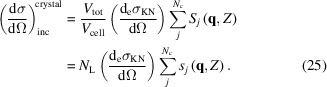



### 2.4. Total scattering   

Using equations (23) and (25), and in analogy with equation (9) for the atomic differential cross section, we write the differential cross section of a small crystal as
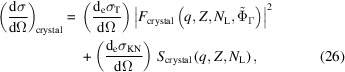
with 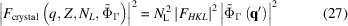
and 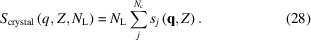
In the coherent scattering term of a crystal (*F*_crystal_), the structure factor *F*_*HKL*_ is analogous to the atomic form factor for the atomic case and dependence on the atomic positions is through the crystal structure factor. It also depends on the number of lattice points and the crystal shape. The incoherent term also depends on the total number of scatterers but it does not depend on the crystal shape or the atomic positions.

## 3. Interference fringes, background and resolution   

### 3.1. General considerations   

In coherent diffraction imaging (CDI), the real-space shape function of an object can be retrieved from its diffraction pattern using iterative phase retrieval algorithms (Miao *et al.*, 1999 ▸). Bragg CDI exploits the diffraction patterns around Bragg peaks and can provide extra information about the strain field in a crystal (Robinson *et al.*, 2001 ▸; Vartanyants & Robinson, 2001 ▸). For samples not prone to radiation damage, the spatial resolution that can be achieved depends crucially on Δ*q*_max_, the extent in reciprocal space of the diffraction pattern that is measured. The best achievable spatial resolution is δ*r* ≃ 2π/Δ*q*_max_ (Stagl *et al.*, 2014 ▸). But to be measurable, the interference fringes must be above the noise level. The contrast of the fringes (*i.e.* their sharpness or distinctness) is often quantified by their visibility, defined as (Born & Wolf, 1999 ▸)
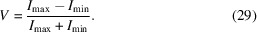
It is in general difficult to predict the visibility of the fringe patterns that will be obtained in an experiment (Yan *et al.*, 2016 ▸) and analytical formulas exist for simple cases only. In the presence of a background signal, *I*_min_ > 0 and the fringe visibility is degraded. A visibility reduction of 50% already occurs when the signal between the fringes, *I*_min_, is *I*_min_= (1/3)*I*_max_. It has already been shown that a reduction in contrast due to partial coherence affects the reconstruction algorithms (Vartanyants & Robinson, 2001 ▸). If the background is high, the fringes may eventually disappear under the noise level. This is what will ultimately limit the maximum the achievable resolution (Stagl *et al.*, 2014 ▸). We will use the signal-to-noise ratio instead of the visibility as a criterion to evaluate when the fringes are below the noise level and obtain the resolution limit.

### 3.2. Noise: a simple model   

We examine the simplest case where the total intensity is the sum of three terms: (*a*) the interference fringes, arising from the coherent scattering (*I*_coh_); (*b*) the featureless background due to Compton scattering (*I*_inc_); (*c*) a noise term (ℵ): 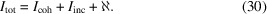
We consider that the total intensity is low and that the noise obeys Poisson statistics. This is justified since we are examining weak interference fringes and signals. The standard deviation of the Poisson noise is (*I*_coh_ + *I*_inc_)^1/2^ (Hughes & Hase, 2009 ▸). In our model, the background due to Compton scattering affects principally the noise level – in the absence of noise, and with a constant background, the coherent contribution could still be extracted by removing the baseline (background) contribution. The signal-to-noise ratio (SNR) is defined as the ratio between the mean value and standard deviation of the signal (Russo, 2018 ▸), 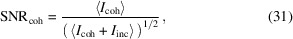
where we have included the subscript ‘coh’ to indicate that we are considering the ratio of coherently scattered intensity to the total noise. The angular brackets denote the mean value. In the following, we consider that the coherent and incoherent contributions are constant and we remove the angular brackets for notation simplicity. The purely coherent contribution is above the noise level (SNR_coh_ ≥ 1) if *I*_coh_ ≥ (*I*_coh_ + *I*_bck_)^1/2^. Using equations (10), (23) and (25), the inequality can be written as
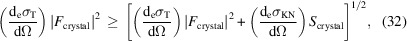
or, equivalently, 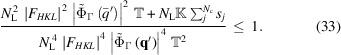


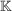
 and 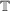 are the KN [equation (3)] and Thomson [equation (7)] differential cross sections, respectively. In a small region of the reciprocal space, the incoherent and coherent scattering functions of the crystal can be considered to be constant, as well as the KN and Thomson differential scattering cross sections. The possible values of the number of unit cells and the normalized shape function lie within the intervals *N*_L_ ∈ [1, ∞) and 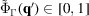, respectively. As the denominator in equation (33) goes as 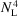, the inequality will always be fulfilled for large enough *N*_L_, that is, for large enough crystals. For a crystal, the best achievable resolution depends on the FT of its shape function. Notably, the ultimate resolution may be different along different 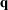 directions because the normalized shape function 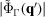 hinges on 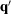.

### 3.3. Analytical solution for the number of unit cells   

It is possible to obtain an analytical expression to compute *N*_L_, the number of unit cells that a crystal should have, so that an *n*th secondary maximum of the normalized shape function (*i.e.* the *n*th interference fringe) is above the noise level and hence can be measured. For that, we rewrite equation (33) as a cubic function of *N*_L_ and we calculate its roots. From equation (33), we write
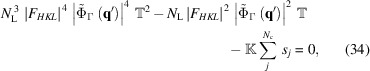
which can be rearranged to obtain a cubic equation of the form 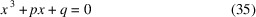
with the following identities,
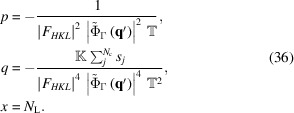
The general solution of an equation of the type of equation (35) is given by the Tartaglia-Cardano’s formula (Aleksandrov *et al.*, 1999 ▸),
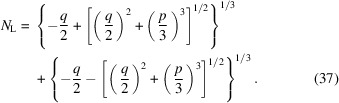
To obtain the number of unit cells that are necessary to be above the noise level, for a given secondary maximum, one needs to introduce in equation (37) the value of the shape function at the secondary maximum, *i.e.* 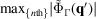. Equation (37) can also be employed to determine, for a given value of the normalized shape function, when the signal starts to be below the noise level, independent of whether it is a secondary maximum or not. Note, however, that the identities for *p* and *q* in equation (36) are undefined when 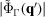 = 0.

### 3.4. Analytical solution for the magnitude of the shape function   

For completeness, we also give the solution for the magnitude of the shape function, although it is of less practical interest. Similar arguments can be used to obtain an analytical expression for 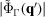 as a function of *N*_L_. Equation (34) is a quartic polynomial on 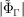, but, by setting 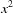 = 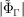, one obtains a quadratic equation whose solution is
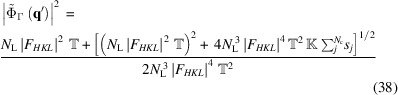
for which we have selected the positive root of the general solution of a quadratic equation because 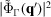 must be positive.

## 4. Practical examples   

We discuss the influence of Compton scattering for some simple cases that are often encountered in diffraction experiments and have analytical solutions. We use the result of equation (37) and analyse in detail the case of a crystal with rectangular shape. Based on that result, we comment on the case of a cylindrical crystal, a regular lattice and a crystal of any shape. We consider a vertical scattering geometry and that in all cases the crystals are illuminated with a beam having a coherence length larger than the crystal dimensions.

### 4.1. Rectangular crystal   

**Figure 3:**
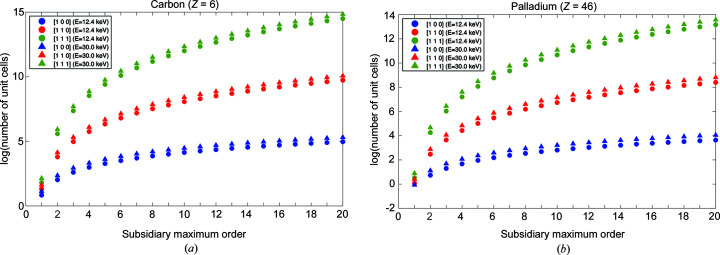
Number of lattice points needed, for a secondary maximum, to be above the noise level versus secondary maximum order. The values have been calculated using the differential scattering cross sections for a 45° scattering angle, for the corresponding energy. [100], [110] and [111] denote three directions (see text for details).

For a crystal with rectangular shape, with sides 2ξ_*x*_, 2ξ_*y*_, 2ξ_*z*_ along the directions *x*, *y*, *z*, respectively, the interference fringes are given by a function of the form (Patterson, 1939 ▸; Krivoglaz, 1996 ▸)
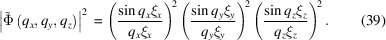
The function 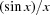 is called the ‘sinus cardinal’ or ‘sinc’ function and it fulfils 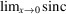 = 1. The sinc^2^ function has subsidiary maxima whenever *x* = π/2. Due to the product of the sinc^2^ functions along the *x*, *y*, *z* directions (that we name here in the following [100], [110] and [111], respectively), those maxima off the principal axes (*i.e.* two or more of *x*, *y* or *z* are non-zero) quickly become much weaker. Equation (37) gives the number of unit cells (*N*_L_) that are necessary so that the secondary maxima along the different directions are above the noise level. The results are given in Table 1 ▸ and shown graphically in Fig. 3 ▸. The values have been calculated using the differential scattering cross sections for a 45° scattering angle for carbon and palladium, at two energies: *E* = 12.4 and 30.0 keV. These particular chemical elements have been chosen for the numerical calculations because carbon is often used as a matrix element where crystallites are embedded, and palladium is an archetypal component of catalytic particles, for which Bragg CDI has already been employed (Ulvestad *et al.*, 2017 ▸). A scattering angle of 45° has been chosen because at 12.4 keV the coherent and incoherent cross sections are roughly equal for carbon and so that the values obtained can serve as an estimate.

The total number of unit cells can be converted into crystal volume using equation (24). Heuristic calculations can be made considering a cubic unit cell of side *a* and a cubic crystal of side ξ, for which *V*_tot_ = ξ^3^ yields a lateral side 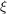 = 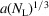, in unit-cell lengths: as a rule of thumb, the lateral size goes as the cubic root of the total number of unit cells times the unit-cell length. For example, taking the data in Table 1 ▸ corresponding to Pd at 12.4 keV and *a* ≃ 0.2 nm, the fifth maximum along the [100] direction is above the noise if ξ > 0.8 nm, along [110] if ξ > 9.4 nm, and along [111] if ξ > 98.2 nm. Thus, the maxima along some directions are above the noise level only for rather large crystals. As mentioned above, the maximum achievable resolution along the different directions has a strong dependence on the crystal shape and size.

### 4.2. Cylindrical crystal   

For a cylinder-shaped crystal that has the cylinder long axis along the *Z*-direction and the circumference of radius *r* on the *XY* plane, the intensity interference fringes are given by the function 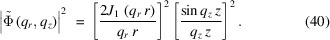

**Figure 4:**
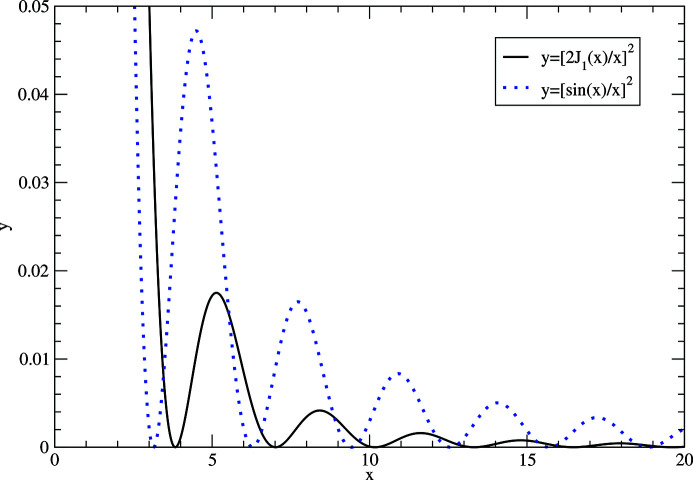
First subsidiary maxima of the Airy function *y* = 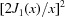 (black solid) and the *y* = 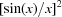 function (blue dotted). For both functions, the value of the principal maximum at *x* = 0 is equal to 1.

*J*_1_(*x*) is a Bessel function of first kind and first order. In the *z* = 0 plane, equation (40) yields an Airy diffraction pattern [*i.e.* the diffraction pattern expected from a circular aperture (Born & Wolf, 1999 ▸)]. The subsidiary maxima of an Airy pattern are also much weaker than the principal maximum (Fig. 4 ▸ and Table 2 ▸). The third subsidiary maximum already is ∼2 × 10^−3^ weaker than the principal maximum. Compared with the sinc function discussed in Section 4.1, the secondary maxima of the Airy function are also weaker, and thus larger crystals are needed to access the secondary maxima.

### 4.3. Regular lattice   

In the derivation of the formulas of Section 2.3.1, there is an implicit assumption: the crystal size is much larger than the lattice spacing. For a small crystal, this approximation breaks, and it is better suited to use directly a sum of the lattice points instead of a FT transform of the crystal shape. The (coherent) scattering amplitude of the crystal is proportional to functions of the form 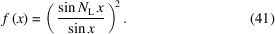
But because 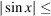 1, the rectangular parallelepipedal crystal case of Section 4.1 can be considered as the limiting case of the regular lattice sum.

### 4.4. Crystal of any shape   

Arbitrary shapes can be approximated by considering polygonal shape functions instead. Analytical formulas for arbitrary polygonal shape functions are derived in the literature (Lee & Mittra, 1983 ▸; Saga, 1987 ▸; Chu & Huang, 1989 ▸; McInturff & Simon, 1991 ▸). The reader is referred to those references, and especially to Chu & Huang (1989 ▸), where a rather simple and easily computable formula is reported. Following the procedure depicted in Section 4.1, it is possible to estimate the maximum resolution that can be achieved for a given polygonal shape. However, there is not a general method and the calculations have to be done for each case.

## 5. Discussion   

It has already been discussed in the literature how noise affects the phase retrieval methods and the strategies that can be adopted to minimize its impact on the inversion schemes (Vartanyants & Robinson, 2001 ▸; Godard *et al.*, 2012 ▸). Our study is focused on determining whether Compton scattering affects the resolution. We have shown that is indeed the case: Compton scattering degrades the visibility of the interference fringes and can even make them disappear under the noise if the inequality in equation (33) is satisfied. The limit dictated by the inequality is, obviously, dependent on the noise model we have used in equation (30) (*i.e.* just counting statistics or Poisson noise). Our noise model is similar to the Rose model and seems justified for low counting statistics (Burguess, 1999 ▸), which is most often the case in BCDI experiments. We have also ignored the origin of the noise; terms arising from detection noise or stray background could be included. A different noise model would yield a different inequality. But, in any case, Compton scattering imposes a resolution limit on the BCDI technique. In principle, using detectors with sufficient energy resolution, one could discriminate between the coherently scattered (elastic) contribution and the Compton (inelastic) scattered part. We note, however, that, even for 25 keV at 180°, the shift in energy is less than 10%. The energy resolution of the hybrid pixel detectors – nowadays the most common detectors used in BCDI – is not enough to set the coherent and incoherent contributions apart (Ballabriga *et al.*, 2016 ▸). Interestingly, a new technique – scanning Compton X-ray microscopy – that uses the coherently and incoherently scattered photons has been proposed. Potentially, it can provide images with better resolution than those obtained with other coherent imaging techniques, and exposing the samples to a lower X-ray dose (Villanueva-Perez *et al.*, 2018 ▸).

We have used a signal-to-noise value of SNR = 1 to set the inequality equation (33) and decide when the diffraction fringes can no longer be discerned. Based on that, a value for the maximum resolution attainable can be established. The criterion SNR = 1 is very low; generally, higher values of the SNR value are needed to detect a signal coming from an object in a noisy and homogeneous background (Russo, 2018 ▸). Rose estimated that the SNR had to be above ∼5 – see Burguess (1999 ▸) for a detailed discussion and the original references. Roughly, such value implies that the total number of unit cells within the crystal should be 25 times larger, or, approximately, that the lateral size of the crystals must be ∼3 times larger than the values calculated with equation (37). We mention that the Rose criterion has also been considered to assess the limits in resolution in X-ray diffraction microscopy due to X-ray radiation damage (Howells *et al.*, 2009 ▸).

We have also shown how, in the presence of noise, the maximum achievable resolution depends upon the crystal shape and the reciprocal space directions probed; this has not been (to our knowledge) brought up before. A direct consequence is that to study small crystallites embedded in an amorphous matrix one should select the matrix material sensibly and also use a beam size that is close to the crystallite size in order to reduce the Compton scattering, even in Bragg geometry at high angles. It is also of special importance for the reconstruction of objects in 3D. To obtain a full data set, one needs to measure 3D data in reciprocal space, generally done by rotating the sample around specific axes and recording rocking scans (Stagl *et al.*, 2014 ▸). In projections along the non-principal directions, the maximum achievable resolution may be smaller because the secondary maxima are much weaker. For strained crystals, the situation may be worse because the diffraction pattern about Bragg peaks is not symmetric: strain within the crystal (*i.e.* 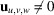) yields an asymmetric contribution to the scattering amplitude around each reciprocal lattice point (Robinson *et al.*, 2001 ▸; Krivoglaz, 1996 ▸). An effect of strain is that in most cases it will smear the interference oscillations arising from coherent scattering. The ratio between maxima and minima may decrease, and the coherent signal will be closer to the background level: the weakest oscillations may vanish, making it more difficult to obtain quantitative information about the strain from the data. Compton scattering differentiates from random noise, in the sense that it is inherent to the sample composition and the scattering geometry. From the point of view of phase retrieval, this does not make any difference. The option of a careful estimation of the Compton contribution to subtract from the data should, in our opinion, be considered with great prudence. More effective could be the reduction of the source of noise by judicious experimental choices.

It is interesting to mention that in small crystals with large unit cells (for example, some protein crystals or metal-organic frameworks), as pointed out by Ewald (1940 ▸), the internal crystalline structure can enhance the interference fringes arising from the shape. Essentially, if the unit cell is very large, the reciprocal space Bragg points due to the inner crystalline structure are very close. However, the Compton scattering remains constant as it is incoherent and therefore independent of the crystalline structure. This enhancement could eventually be beneficial for studies on small crystals with large unit cells. We note nevertheless that if overlap of shape functions centred at adjacent reciprocal space points occur, using a CDI-type strategy may be difficult or impossible, because it will be difficult to set apart the shape function.

How relevant are our findings in practice? We examine two recent works on BCDI to ascertain it, focusing only on the aspects that are pertinent for our comparison. Ulvestad *et al.* obtained 3D imaging results on Pd nanocrystals during the hydriding phase transformation (Ulvestad *et al.*, 2017 ▸). The BCDI experiment was carried out using 9 keV X-rays, at 2Θ = 33°. The smallest particles they studied had an effective length (*i.e.* *l* ≃ volume^1/3^) of *l* ≃ 180 nm. Their images show secondary oscillations up to 17 secondary maxima and probably more could be measured with longer exposure times. If these particles are considered, in a very coarse approximation, as small spheres, the FT shape function would be proportional to the square of the *f*(*r*) = 3[sin(*ql*)/(*ql*)^2^ − cos(*ql*)/(*ql*)^2^] function, where *l* denotes the radius of the sphere. This function is connected with the Bessel functions [see Vembu (1961 ▸) for details]. It is very compelling that, compared with the case of the rectangular crystal depicted in Section 4.1 for which crossed multiplicative terms decrease the signal diffracted along non-principal directions, a spherical particle yields a less marked intensity decrease along any direction except when compared with the directions perpendicular to the rectangle faces. One can conclude that for spherical or spheroidal particles, Compton scattering, and by extension any noise in general, will have a smaller influence on the spatial resolution that could be achieved. These considerations apply chiefly to non-strained crystals. A more detailed study would be required to quantify to which extent they are also valid (or not) for strained crystals. With respect to the work of Ulvestad *et al.*, the beam energy, scattering angle and crystal size are more favourable than those used to obtain the values in Table 1 ▸. Evaluating equation (37), the number of unit cells that are required for the 20th secondary maximum to be visible is ∼16 nm, a size substantially smaller than the crystals studied by Ulvestad *et al.*. Thus, unless very small particles are studied, for most of the studies that are performed at rather low energies (∼8 keV), we do not anticipate that Compton scattering will lessen the resolution noticeably.

The second work we comment on is a recent single-shot 3D CDI experiment in which core-shell cubic Au/Pd nanoparticles have been imaged (Pryor *et al.*, 2018 ▸). The experiment was done in forward scattering geometry using 6 keV X-rays, and therefore Compton scattering is completely negligible. One of the ultimate aims of such experiments is to characterize the internal structure and quantify the stress relaxation mechanisms. Large-scale molecular dynamics calculations have been employed to simulate the core-shell Au/Pd particles (Nathanson *et al.*, 2018 ▸). It is predicted that strain in the palladium shell is larger on the faces than at the edges and corners. There is an interest in determining these differences as they may influence the optical and electrochemical properties of the core-shell particles. However, to measure the strain one has to resort to, for example, BCDI, and measure the diffracted signal at larger scattering angles. Moreover, directions different to the principal ones (*i.e.* perpendicular to the cube faces) must be examined too to probe the strain at edges and corners. As discussed in Section 4.1, along the non-principal directions the effect of Compton scattering will be more noticeable because the coherently scattered signal is much weaker. This is also evident in the images reported by Pryor *et al.* According to our calculations (Table 1 ▸ and Section 4.1), at 12.4 keV and 2Θ = 45° scattering angle, the fifth secondary maximum along the [111] direction of a palladium cystallite would already be compromised for sizes smaller than ∼98 nm. Hence, Compton scattering may bar these studies and limit their resolution. But, notwithstanding the unavoidable Compton scattering, clever approaches, such as the super-resolution 3D CDI technique employed by Pryor *et al.* (2018 ▸), may push the ultimate resolution well beyond the limit of a ‘classic’ approach. Our results are circumscribed to the standard BCDI approach and are not directly applicable to super-resolution approaches, although they may also be relevant to those methods too, but it is difficult to evaluate to what extent.

A final note regarding partial coherence and Compton scattering: the effect of the incoherent contribution of Compton scattering from the sample is different from that arising from an incoming partially coherent beam. In the latter case, the incoming wavefield can be characterized by measuring directly the incoming beam. That information can be taken into account in the phase retrieval algorithms (Mastropietro *et al.*, 2011 ▸; Björling *et al.*, 2020 ▸). However, the loss of coherence due to Compton scattering from the sample and which will be observed on the total scattering signal cannot be easily input into a phase retrieval strategy without having some prior knowledge about the sample.

## 6. Conclusions   

Compton scattering may limit the resolution of BCDI by reducing the visibility of the interference fringes. Certainly, its influence will be greater when the Compton scattering cross section is larger: for higher energies and for systems with low-*Z* elements. It will also be more important for small crystals. Its relative importance can be estimated using a criterion that relates the signal-to-noise ratio, the crystal size (or number of lattice points) and the shape function [equation (33)]. Notably, the resolution will be limited for those directions where the shape function interference fringes are fainter.

## Figures and Tables

**Table 1:** Number of lattice points (*N*_L_) that are required for the *n*th secondary maxima along the [100], [110] and [111] directions to be above the noise level The calculations have been performed for two energies (*E* = 12.4 and 30.0 keV) and using the differential scattering cross sections for 45° scattering angle, for carbon (C) and palladium (Pd).

	*N*_L_					
	[100]		[110]		[111]	
Maximum order	C	Pd	C	Pd	C	Pd
Energy = 12.4 keV						
1	7	0	2.100 × 10^1^	1.000 × 10^0^	6.400 × 10^1^	3.000 × 10^0^
2	107	5	6.285 × 10^3^	2.960 × 10^2^	3.894 × 10^5^	1.812 × 10^4^
3	403	20	9.522 × 10^4^	4.440 × 10^3^	2.316 × 10^7^	1.077 × 10^6^
4	977	47	5.721 × 10^5^	2.662 × 10^4^	3.417 × 10^8^	1.588 × 10^7^
5	1898	90	2.185 × 10^6^	1.016 × 10^5^	2.552 × 10^9^	1.186 × 10^8^
6	3230	153	6.370 × 10^6^	2.961 × 10^5^	1.271 × 10^10^	5.906 × 10^8^
7	5030	237	1.552 × 10^7^	7.217 × 10^5^	4.836 × 10^10^	2.247 × 10^9^
8	7355	346	3.330 × 10^7^	1.548 × 10^6^	1.519 × 10^11^	7.061 × 10^9^
9	10257	481	6.492 × 10^7^	3.017 × 10^6^	4.135 × 10^11^	1.922 × 10^10^
10	13786	646	1.175 × 10^8^	5.460 × 10^6^	1.007 × 10^12^	4.679 × 10^10^

Energy = 30.0 keV						
1	14	1	4.300 × 10^1^	3.000 × 10^0^	1.360 × 10^2^	8.000 × 10^0^
2	227	13	1.368 × 10^4^	7.540 × 10^2^	8.502 × 10^5^	4.664 × 10^4^
3	869	49	2.078 × 10^5^	1.141 × 10^4^	5.059 × 10^7^	2.774 × 10^6^
4	2116	117	1.249 × 10^6^	6.853 × 10^4^	7.465 × 10^8^	4.093 × 10^7^
5	4121	228	4.772 × 10^6^	2.617 × 10^5^	5.574 × 10^9^	3.056 × 10^8^
6	7022	387	1.391 × 10^7^	7.630 × 10^5^	2.776 × 10^10^	1.522 × 10^9^
7	10947	603	3.391 × 10^7^	1.859 × 10^6^	1.056 × 10^11^	5.792 × 10^9^
8	16018	882	7.274 × 10^7^	3.989 × 10^6^	3.319 × 10^11^	1.820 × 10^10^
9	22348	1230	1.418 × 10^8^	7.775 × 10^6^	9.033 × 10^11^	4.953 × 10^10^
10	30047	1653	2.566 × 10^8^	1.407 × 10^7^	2.199 × 10^12^	1.206 × 10^11^

**Table 2:** First ten subsidiary maxima of the [{{2J_{1}(x)}/{x}}]^{2} function

Maximum order	*x*	[{{2J_{1}(x)}/{x}}]^{2}
1	5.14	1.750 × 10^−2^
2	8.42	4.158 × 10^−3^
3	11.62	1.601 × 10^−3^
4	14.80	7.794 × 10^−4^
5	17.96	4.370 × 10^−4^
6	21.12	2.693 × 10^−4^
7	24.27	1.776 × 10^−4^
8	27.42	1.232 × 10^−4^
9	30.57	8.896 × 10^−5^
10	33.72	6.633 × 10^−5^
